# Experience with exercise right heart catheterization in the diagnosis of pulmonary hypertension: a retrospective study

**DOI:** 10.1186/2049-6958-9-51

**Published:** 2014-10-15

**Authors:** Stephan Keusch, Anina Bucher, Séverine Müller-Mottet, Elisabeth Hasler, Marco Maggiorini, Rudolf Speich, Silvia Ulrich

**Affiliations:** Clinic of Pulmonology, University Hospital of Zurich, 8091 Zurich, Switzerland; Clinic for Internal Medicine, University Hospital of Zurich, Zurich, Switzerland; Zurich Center for Integrative Human Physiology, Zurich, Switzerland

**Keywords:** Exercise, Pulmonary arterial hypertension, Pulmonary hypertension, Right heart catheterization

## Abstract

**Background:**

Data on exercise pulmonary hemodynamics in healthy people and patients with pulmonary hypertension (PH) are rare. We analyzed exercise right heart catheterization (RHC) data in a symptomatic collective referred with suspected PH to characterize the differential response by diagnostic groups, to correlate resting with exercise hemodynamics, and to evaluate safety.

**Methods:**

This is a retrospective single-center study reviewing data from patients in whom an exercise RHC was performed between January 2006 and January 2013. Patients with follow-up RHC under PH -therapy were excluded.

**Results:**

Data from 101 patients were analyzed, none of them had an adverse event. In 35% we detected a resting PH (27.8% precapillary, 6.9% postcapillary). Exercise PH (mean pulmonary arterial pressure (mPAP) >30 mmHg at exercise) was found in 38.6%, whereas in 25.7% PH was excluded. We found a remarkable number of exercise PH in scleroderma patients, the majority being postcapillary. 83% of patients with mPAP-values between 20 and 24.9 mmHg at rest had exercise PH. Patients with resting PH had worse hemodynamics and were older compared with exercise PH ones.

**Conclusion:**

In this real-life experience in symptomatic patients undergoing exercise RHC for suspected PH, we found that exercise RHC is safe. The facts that the vast majority of patients with mPAP-values between 20 and 24.9 mmHg at rest had exercise PH and the older age of patients with resting PH may indicate that exercise PH is a precursor of resting PH. Whether earlier treatment start in patients with exercise PH would stabilize the disease should be addressed in future studies.

## Background

Pulmonary hypertension (PH) is defined as a mean pulmonary artery pressure (mPAP) ≥25 mmHg during resting right heart catheterization (RHC) in supine position for at least 15 minutes [1]. PH may occur in association with various diseases, but also without any obvious reason (idiopathic). PH has been classified by the World Health Organization (WHO) in 5 classes, namely pulmonary arterial hypertension (class I), PH owing to left heart disease (class II), pulmonary hypertension due to lung diseases and/or hypoxia (class III), chronic thromboembolic PH (class IV) and PH with unclear multifactorial mechanisms (class V) [2]. PH can only be safely diagnosed invasively by right heart catheterization. Hereby, precapillary PH can be separated from pulmonary venous hypertension by measurement of the pulmonary artery wedge pressure (PAWP).

The cardinal symptom of patients suffering from all forms of PH is exertional dyspnea. This is the reason why the condition is often diagnosed late in its clinical course. Most patients feel well at rest, however, increasingly minor exercise poses a major problem to the affected. Until recently, PH was not only defined as mPAP ≥ 25 mmHg at rest, but also as >30 mmHg at exercise [3–5]. However, to date only few data supporting this definition is available, and the normal response to exercise of healthy subjects, especially of elderly, is not well known. Thus, the definition of exercise-induced PH was cancelled during the WHO meeting in Dana Point, 2009 and not re-introduced in Nice, 2013 [1].

At our PH center at the University Hospital of Zurich, measurement of pulmonary hemodynamics during exercise by RHC, using a cycle ergometer in supine position, is performed since 2005.

As data on exercise hemodynamics in healthy people is mostly available from relatively old studies and data on PH-affected patients is rare [6, 7], we aimed to retrospectively analyze all exercise RHC performed at our center in order to correlate resting with exercise hemodynamics, to characterize the differential response to exercise in the various PH classes, and to investigate the value of exercise RHC in the upmost mPAP range of normal (from 20 to 24.9 mmHg) formerly labelled as borderline PH. A further aim was to look at the safety of exercise RHC procedure.

## Methods

### Study design and study subjects

**Figure 1:**
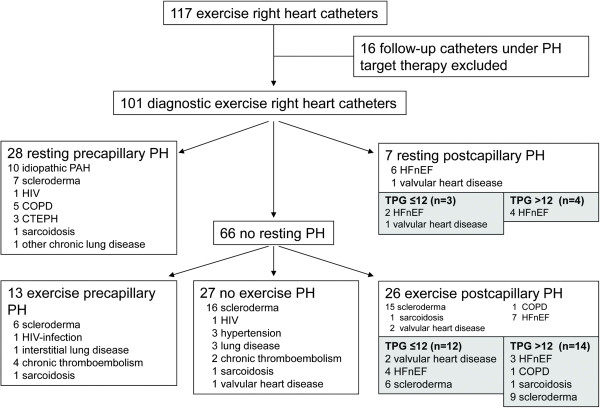
**Patients and diagnostic groups after right heart catheterization.** HFnEF, heart failure with normal ejection fraction, PAH, pulmonary arterial hypertension; PH, pulmonary hypertension; COPD, chronic obstructive pulmonary disease; CTEPH, chronic thromboembolic pulmonary hypertension; TPG, transpulmonary pressure gradient.

This is a retrospective, observational study performed at our referral center for PH. We reviewed all RHC data from January 2006 until January 2013. Eligible patients were all those in whom a RHC with additional recording of exercise hemodynamics was performed. From the total 117 eligible patients we excluded 16 patients because they were already taking PH specific medical therapy and the exercise RHC was performed in the follow up after establishing appropriate targeted therapy (Figure 1). All patients included in the study (n = 101) were referred with clinical suspicion of PH, mostly because of exertional dyspnea or progressive diffusion limitation for carbon monoxide in scleroderma patients, and a diagnostic RHC at rest and during exercise was performed.

### Methods

By reviewing the medical histories we collected the hemodynamic data and patients’ characteristics (demographics, vital signs, PH class according to Dana Point classification scheme, 6 minute walk distance, and functional limitation [WHO/NYHA class]). In addition, we looked for possible adverse events during or shortly after the RHC procedure and gathered follow up data regarding survival by clinical medical records or contacts to the treating physicians. RHC was performed in supine position using a conventional pulmonary artery catheter and Edwards Vigilance Monitor for cardiac output measuring (continuous cardiac output thermodilution catheter). The usual invasively measured hemodynamic parameters were recorded. Baseline measurements were recorded after 15 minutes of rest and repeated 5 to 10 minutes later. For further calculations the mean of these resting values was taken. For the exercise testing we used a dynamic, symptom limited, step-wise incremental cycling exercise protocol in supine position. Patients were challenged by a cycle ergometry (TheraVital, Medica GMbH, Ravensburg) in supine position starting at 10 watts. The work load was increased every 3 minutes by 10 watts until symptoms occurred that lead to termination of exercise or in total 15 minutes of exercise was achieved. Data were recorded every 3 minutes before increasing the work load.

The patients were allocated to different diagnostic groups according to their resting hemodynamics and their hemodynamic response to exercise. The following groups were formed: (1) resting precapillary PH (mPAP ≥ 25 mmHg, PAWP < 15 mmHg; PAH group), (2) exercise precapillary PH (mPAP during exercise > 30 mmHg, PAWP < 15 mmHg; ePAH group), (3) resting postcapillary PH (mPAP ≥ 25 mmHg, PAWP >15 mmHg; PVH group), (4) exercise postcapillary PH (mPAP during exercise > 30 mmHg, PAWP > 15 mmHg; ePVH), (5) exercise out-of-proportion PH (mPAP during exercise > 30 mmHg, PAWP > 15 mmHg, transpulmonary gradient (TPG) > 12; eoPVH group) and (6) no PH (neither at rest, nor at exercise).

### Statistical analysis

All baseline data are summarized by medians and quartiles. Baseline variables by groups were compared using Mann-Witney-U-Test. P <0.05 was considered significant. Statistica 10 (StatSoft, Inc., Tulsa, USA), SPSS 19 and Microsoft Excel (Version 2010) were used.

### Ethics

All patients gave their written informed consent to register their data for scientific purposes and the study was approved by the local ethical authorities (KEK 2012-0125).

## Results

A total of 101 patients with exercise performance during RHC were included. They all underwent catheterization because of clinical suspicion of PH, mostly due to exertional dyspnea or progressive diffusion limitation for carbon monoxide in scleroderma patients. The overall baseline characteristics of these patients are shown in Table 1 and 2. According to the hemodynamic characteristics established during RHC the included patients were allocated to the different groups as described in the methods section. The absolute numbers in each group are listed in Figure 1. About one third of patients had resting PH (PAH or PVH). Out of the two thirds of patients without resting PH 13 patients (20%) suffered from ePAH, 26 had ePVH (39%), and 27 patients (41%) did not have elevated pulmonary pressures at rest or exercise. A high proportion of scleroderma (n = 15, 34% of all included scleroderma patients) were in the group of ePVH.

**Table 1:** **Baseline characteristics**

Total number of patients, n (%)	101
Female, n (%)	70 (69.3%)
Age, years	61 (52;68)
**Relevant clinical diagnosis before Catheter-assessment, n (%)**	
Scleroderma	44 (44%)
Chronic lung disease	11 (11%)
History of pulmonary embolism	9 (9%)
Sarcoidosis	4 (4%)
HIV	3 (3%)
Other unclear dyspnea	30 (30%)
**NYHA/WHO functional class, n (%)**	
Class I/II	11 (11%)/35 (35%)
Class III/IV	42 (41%)/13 (13%)
Body mass index, kg/m2	25.1 (21.9;28.2)
6 minute walking distance, m	475 (335;539)

Data given as median (quartiles) or number (%). NYHA: New York Heart Association, WHO: World Health Organization.

**Table 2:** **Patients characteristics according to diagnostic groups**

Diagnostic group	Resting precapillary PH, PAH (n = 28)	Exercise precapillary PH, ePAH (n = 13)	Resting postcapillary PH, PVH (n = 7)	Exercise postcapillary PH, ePVH (n = 12)	Exercise postcapillary out-of-proportion PH, eoPVH (n = 14)	No PH (n = 27)
Age, years	63.7^¶^ (58.6; 70.4)	53.9 (51.8; 62.6)	73.4^¶^ (64.3; 73.8)	61.2 (51.0; 67.4)	61.3 (54.6; 68.3)	53.9 (43.3; 66.0)
Females, n	15 (53.6)	9 (69.2)	6 (85.7)	8 (66.7)	9 (64.3)	23 (85.2)
BMI, kg/m2	25.3^¶^ (22.3; 27.8)	25.9 (23.2; 28.1)	28.0^¶^ (24.4; 30.2)	25.1 (21.8; 27.8)	27.3^¶^ (25.3; 29.7)	22.8 (18.7; 25.4)
NYHA class II/III/IV, n	28 (100)	12 (92.3)	7 (100)	10 (83.3)	12 (85.7)	21 (77.8)
6MWD, m	459.5 (296; 504)	500 (414; 552)	437 (339; 450)	566 (460; 603)	490 (330; 532)	450 (300; 540)
SpO_2_ after 6MWD, %	90 (80; 93)	93.5 (82; 96)	95 (95; 96)	96 (94; 98)	94 (89; 96)	94 (89; 95)
HR after 6MWD, bpm	109 (91; 127)	104 (83; 115)	98 (88; 109)	104 (91; 116)	118 (108; 137)	103 (100; 120)
SBP after 6MWD, mmHg	138 (120; 161)	131 (120; 142)	175 (154; 198)	130 (116; 154)	155 (140; 160)	139 (138; 158)

Data given as median (quartiles) or number (%). ^¶^= p <0.05 compared to patients with no PH. No significant differences between exercise postcapillary PH and exercise postcapillary out-of-proportion PH were detected.

6MWD, Six minute walk distance, *BMI,* Body mass index; ePAH*,* Exercise precapillary pulmonary hypertension; eoPVH, Exercise postcapillary out-of-proportion pulmonary hypertension; *ePVH*, Exercise postcapillary pulmonary hypertension; *HR*, Heart rate; *NYHA*, New York Heart Association, *PAH*, Pulmonary arterial hypertension; *PH*, Pulmonary hypertension; *PVH*, Postcapillary pulmonary hypertension; *SpO*_*2*_, Peripheral oxygen saturation; *SBP,* Systemic blood pressure.

A separate clustering was done with patients that presented with mPAP from 20 to 24.9 mmHg. A total of 29 patients fitted according to their hemodynamics in this group. A high proportion of these patients (n = 24) showed an elevation of mPAP above 30 mm Hg during exercise (83%). Eight patients presented with ePAH (28%) and 16 patients presented with ePVH (55%), only 5 patients in this cluster had no PH at exercise (17%).

A total of 37 patients had mPAP< 20 mmHg. Intriguingly 15 patients in this group presented with exercise PH forms (40.5%); namely 5 with ePAH, and 10 with ePVH.

Comparisons between the different PH groups show-ed mainly a greater variance in the pulmonary hemo-dynamics in the groups of resting PH, either precapillary or postcapillary (see Table 3). In precapillary PH groups we found a greater increase in mPAP during exercise in PAH compared to the ePAH group. Accordingly, also right atrial pressure and pulmonary vascular resistance were higher, arterial oxygen saturation and mixed venous oxygen saturation were lower in the PAH group. The mean blood pressure at exercise was higher in the PVH group compared to non-PH patients. The increase in mPAP was statistically not different in the postcapillary PH groups (PVH versus ePVH). The right atrial pressure and per definition the PAWP were higher in PVH.

**Table 3:** **Hemodynamic data according to diagnostic groups**

Diagnostic group	Resting precapillary PH, PAH (n = 28)	Exercise precapillary PH, ePAH (n = 13)	Resting postcapillary PH, PVH (n = 7)	Exercise postcapillary PH, ePVH (n = 12)	Exercise postcapillary out-of-proportion PH, eoPVH (n = 14)	No PH (n = 27)
**Resting hemodynamics**						
HR, bpm	79 (69; 86.5)	71.5 (66; 83)	79 (74; 84)	70.5^¶^ (61; 74)	76 (70; 83)	78 (70; 86)
Mean BP, mmHg	91 (85; 104)	87 (78; 91)	110 (78; 126)	93 (86; 101)	95 (84; 101)	90 (86; 104)
Mean PAP, mmHg	32^¶,^* (26; 44)	20^¶^ (18; 21)	30^¶,^* (28; 34)	19^**§**^ (15; 22)	22^¶^ (19; 23)	15 (14; 18)
Diastolic PAP, mmHg	23^¶,^* (18; 31)	14^¶^ (12; 15)	21^¶,^* (19; 24)	12 (10; 15)	15^¶^ (13; 18)	11 (10; 14)
RAP, mmHg	8 (4.5; 9.5)	6 (4; 7)	9^¶,^* (7; 10)	5 (3; 8)	6 (4; 9)	5 (4; 7)
PAWP, mmHg	12^¶,^* (10; 13)	8 (8; 11)	17^¶,^* (17; 19)	12 (8; 14)	11^¶^ (9; 12)	8 (7; 10)
TPG, mmHg	23^¶,^* (17; 28)	9^¶^ (9; 12)	13^¶,^* (11; 14)	7^**§**^ (5; 8)	9^¶^ (9; 12)	7 (6; 9)
DPG, mmHg	10^¶,^* (8; 17)	5^¶^ (3; 7)	4 (1; 6)	1^¶,^^**§**^ (0; 3)	3 (2; 6)	2 (1; 3)
CI, l/min/m-2	3.0 (2.5; 3.5)	3.4 (2.8; 3.9)	3.2 (3.0; 4.8)	3.0 (2.75; 3.7)	3.5 (3.0; 4.0)	3.0 (2.7; 4.0)
CO, l/min	5.4 (4.1; 6.7)	6.3 (4.9; 7.2)	6.0 (5.0; 10.8)	5.3 (4.6; 7.0)	6.7^¶^ (5.5; 7.4)	5.1 (4.2; 6.8)
PVR, dynes*s*m-2	330^¶,^* (248; 415)	135 (112; 145)	193 (105; 247)	83^¶,^^**§**^ (63; 95)	122 (97; 175)	109 (90; 158)
SVR, dynes*s*m-2	1296 (1018; 1656)	1026 (898; 1382)	1031 (717; 1875)	1263 (948; 1562)	1196 (1026; 1331)	1340 (1101; 1761)
SaO_2_, %	90.5^¶,^* (87.8; 93.9)	95.0 (93.0; 95.3)	94.0 (93.0; 95.0)	94.8 (92.7; 96.0)	94.8 (93.0; 95.3)	94.8 (93.0; 96.0)
PaO_2_, kPA	8.2^¶,^* (7.6; 9.6)	10.6 (9.2; 11.7)	9.5^¶^ (8.9; 10.00)	10.2 (9.0; 11.7)	10.3 (9.4; 11.4)	11.4 (10.1; 12.2)
PaCO_2_, kPA	4.60 (4.32; 5.35)	4.65^¶^ (4.51; 4.80)	4.70 (4.49; 5.30)	4.85 (4.50; 5.27)	5.13 (4.60; 5.30)	5.05 (4.75; 5.40)
SmvO_2_, %	66.1^¶,^* (60.6; 70.5)	71.0 (69.0; 74.4)	70.0 (65.0; 71.0)	71.6 (68.4; 76.1)	71.4 (69.1; 73.5)	70.0 (65.4; 72.8)
**Hemodynamics during supine maximal cycling exercise**						
Watt achieved	30 (20; 50)	30 (20; 40)	30 (20; 30)	30 (20; 50)	30 (20; 45)	30 (20; 40)
HR, bpm	110 (103; 123)	102 (98; 116)	111 (101; 118)	105 (98; 122)	116.5 (106; 122)	120 (101; 126)
Mean BP, mmHg	115 (101; 125)	107 (92; 114)	128^¶^ (102; 158)	107 (93; 120)	107 (103; 115)	101 (91; 113)
Mean PAP, mmHg	58^¶,^* (50; 66)	35^¶^ (32; 37)	47^¶,^* (42; 65)	36^¶^ (31; 41)	42^¶^ (38; 43)	24 (18; 27)
Increase in mPAP with exercise, mmHg	20^¶,^* (16; 30)	14^¶^(13; 19)	17^¶^(12; 31)	16^¶^(12; 25)	19^¶^ (14.7; 21)	7 (3; 9)
Increase in mPAP per Watt, mmHg	0.73^¶^ (0.53; 1.05)	0.47^¶^ (0.33; 0.74)	0.57^¶^ (0.40; 1.10)	0.48^¶^ (0.36; 0.77)	0.66^¶^ (0.35; 0.85)	0.20 (0.10; 0.33)
Diastolic PAP, mmHg	40^¶,^* (32; 45)	24^¶^ (22; 25)	30^¶^ (26; 40)	27^¶^ (24; 30)	30^¶^ (25; 31)	16 (12; 18)
RAP, mmHg	12^¶,^* (7; 17)	7 (3; 8)	16^¶,^* (9; 18)	10^¶^ (7; 11)	8^¶^ (6; 9)	5 (2; 7)
PAWP, mmHg	16^¶,^* (13; 21)	12 (10; 14)	32^¶,^* (27; 36)	26.5^¶^ (23; 34)	21.5^¶^ (17; 28)	10 (7; 15)
TPG, mmHg	41^¶,^* (34; 45)	22^¶^ (21; 29)	16 (8; 30)	9^¶,^^**§**^ (6; 12)	18^¶^ (16; 21)	12 (9; 14)
DPG, mmHg	21^¶,^* (13; 29)	11^¶^ (10; 13)	1 (-9; 7)	-1.5^¶,^^**§**^ (-6.5; 2.0)	5.5 (3; 9)	4 (2; 6)
CI, l/min/m-2	3.8 (3.0; 4.3)	4.0 (3.7; 4.7)	3.7 (3.3; 5.4)	3.7 (3.2; 4.1)	4.2 (3; 4.8)	3.8 (3.1; 4.3)
CO, l/min	6.8 (5.0; 8.3)	7.2 (6.7; 8.4)	6.5 (5.6; 12.1)	6.5 (5.5; 8.0)	7.06 (6.44; 8.83)	6.4 (4.8; 8.3)
PVR, dynes*s*m-2	487^¶,^* (398; 534)	251^¶^ (207; 335)	163 (100; 198)	97^¶,^^**§**^ (82; 119)	173.5 (148; 227)	139 (107; 229)
Delta PVR, dynes*s*m-2	126^¶^ (68; 208)	103^¶^ (73; 167)	15 (-89; 93)	16^¶,^^**§**^ (-9; 36)	57 (37; 97)	34 (11; 89)
SVR, dynes*s*m-2	1186 (866; 1679)	1031 (907; 1077)	1413 (739; 1541)	1164 (903; 1327)	989.5 (911; 1251)	1222 (933; 1755)
SaO_2_, %	88.0^¶,^* (80.0; 94.0)	95.0 (86.0; 96.0)	96.0 (94.0; 97.0)	94 (93; 95)	93.4^¶^ (91; 94.5)	96.0 (95.0; 97.0)
SmvO_2_, %	36.5^¶,^* (28.2; 43.5)	52.0 (38.0; 55.0)	45.0 (24.0; 50.0)	44.5 (33.5; 57.5)	50 (36; 58)	48.0 (40.0; 55.0)

Data given as median (quartiles) or number (%). ^¶^= p < .05 compared to patients with no PH. *= p < .05 compared to patients with appropriate exercise PH (either resting precapillary PH compared to exercise precapillary PH, or resting postcapillary PH compared to exercise postcapillary PH). ^§^= p < .05 compared to exercise out-of-proportion PH.

BP, Blood pressure; CI*,* Cardiac index; CO, Cardiac output; *DPG*, Diastolic pressure gradient; ePAH, Exercise precapillary pulmonary hypertension; eoPVH, Exercise postcapillary out-of-proportion pulmonary hypertension; *ePVH*, Exercise postcapillary pulmonary hypertension; *HR*, Heart rate; *PaO*_*2*_, Arterial oxygen partial pressure; *PaCO*_*2*_ *,* Arterial carbon dioxide partial pressure; *PAH,* Pulmonary arterial hypertension; *PAP*, Pulmonary arterial pressure; *PAWP,* Pulmonary artery wedge pressure; *PH*, Pulmonary hypertension; *PVH*, Postcapillary pulmonary hypertension; *PVR*, Pulmonary vascular resistance; *RAP*, Right atrial pressure; *SaO*_*2*_, Arterial oxygen saturation; *SmvO*_*2*_, Mixed venous oxygen saturation; *SVR*, Systemic vascular resistance; *TPG*, Transpulmonary pressure gradient.

Comparison between ePVH and eoPVH was performed. The baseline characteristics did not show any statistical differences. The pulmonary hemodynamics were more compromised in the eoPVH group showing a higher mPAP and PVR at rest and a greater increase in PVR during exercise compared to ePVH.

The median follow up for mortality in all patients was 1,024 days (289; 1,287). The length of follow up of each group as well as deaths and adverse events are shown in Table 4. The event rate and group size were too small to establish mortality differences between groups. During performance of RHC with exercise we encountered no adverse events and consider the procedure in experienced hands as safe.

**Table 4:** **Follow up, adverse events and mortality data**

Diagnostic group	Resting precapillary PH (n = 28)	Exercise precapillary PH (n = 13)	Resting postcapillary PH (n = 7)	Exercise postcapillary PH (n = 12)	Exercise postcapillary out-of-proportion PH (n = 14)	No PH (n = 27)
Follow-up days, n	1,080 (246; 1231)	1,031 (366; 1346)	1,080 (279; 1290)	718 (321; 1440)	1,112 (542; 1332)	714 (282; 1241)
Death, n	1	1	1	1	0	2
Lung transplantation, n	3	0	0	0	0	0
Pulmonary endarterectomy, n	2	1	0	0	0	0
Adverse events at RHC, n	0	0	0	0	0	0

A low event rate precludes statistical comparison. PH, Pulmonary hypertension, RHC, Right heart catheterization.

## Discussion

In this observational study of patients with clinical suspicion of PH we found resting PH in 35% of patients. In 38.6% of patients we detected either precapillary (ePAH) or postcapillary exercise PH (ePVH) and in 25.7% of patients PH could be excluded. We found a remarkable number of exercise PH in the scleroderma patients (ePAH or ePVH), a population well-known for its highest risk of developing PH. A great number of ePVH was found in this patient collective. In the cluster of patients with resting mPAP-values between 20 and 24.9 mmHg, 83% showed exercise PH. The groups of resting PH forms showed more severe hemodynamic compromise of the pulmonary vasculature.

The normal pressures in the pulmonary vascular bed at rest and mainly at exercise are still in discussion. In early studies of Wood in the 1950s a normal mPAP of 20 mmHg was estimated [7, 8]. Actual guidelines define PH by a cut-off-value of ≥ 25 mmHg, whereas a recent systematic review showed that the upper limit of normal is about 20 to 21 mmHg [6]. During exercise the physiological rise in pulmonary pressures is intensively debated. Until the WHO consensus guidelines in 2009, it was accepted that a mPAP> 30 mmHg during exercise exceeds the limits of normal [4]. Because of insufficient data on exercise pulmonary hemodynamics, this definition did not find its way in the latest guidelines [1]. A systematic review of historically reported exercise RHC in presumptively healthy people found an age-dependent rise in mPAP during exercise. 21% of healthy persons aged < 50 years reached mPAP-values of > 30 mmHg and 47% of subjects > 50 years showed a mPAP of > 30 mmHg at maximal exercise [6]. In our study of symptomatic patients with a median age of 61 years, including a high proportion of patients at highest risk for PH (scleroderma), nearly 40% of patients reached mPAP-values above 30 mmHg at relatively low exercise levels and we strongly believe that this increase was clinically relevant for the patients’ symptoms. A remarkable large proportion of patients (83%) with mPAP-values between 20 and 24.9 mmHg are found in the exercise PH groups. This fact nourishes the suspicion that this group may suffer from early forms of PH. A signal that even mPAP values < 20 mmHg may already be associated with pathological exercise pulmonary hemodynamic derives from the fact that 40% of the symptomatic patients in our study with a mPAP< 20 mmHg reached values > 30 mmHg during exercise. Also the significant rise of PVR during exercise in the ePAH group versus the no PH group indicates the pathological hemodynamic response of the pulmonary vascular bed during exercise in these patients. We found a significant correlation of the change in PVR with the six-minute walk distance (r = -0.271, p = 0.023 and are thus in line with a meta-analysis by Savarese, who similarly showed a significant correlation of the change in PVR with exercise performance measured by the six-minute walking distance (r = -0.55, p = 0.04). No association with clinical events such as death or hospitalization could be established though [9]. In patients with chronic obstructive pulmonary disease or pulmonary fibrosis resting mPAP values of > 17-18 mmHg were already associated with increased mortality or clinical worsening [10, 11].

The impact of age and its physiological effect on exercise pulmonary hemodynamics is difficult to establish in lack of data in healthy and diseased subjects. Recently, Whyte published data on 38 patients aged < 50 years and free of PH who underwent resting and exercise RHC. Out of these 38 patients, 63% developed mPAP-values > 30 mmHg at mild-to-moderate exercise (< 60 Watt). Compared to healthy subjects not increasing their mPAP above 30 mmHg, those with a significant increase in mPAP showed higher resting mPAP and higher PVR [12]. In another study of symptomatic patients referred for RHC, a greater number showed PVH (48%). From those classified as PAH 84% suffered from ePAH. The mean age of this group was 58.8 years [13]. In a registry analysis of Condliffe in connective tissue disease-associated PAH the proportion of patients with ePAH was 11.4% and about 19% of them progressed within 2 to 3 years to PAH. Progression to PAH in this scleroderma cohort occurred in 60% of patients with ePAH who died in the follow up period of maximal 6 years [14]. We believe that further studies have to shed light on this issue, especially on the age-dependency of cut-off values during exercise.

In our scleroderma subgroup, a collective that is at highest risk for developing PH, we found pre- and postcapillary PH-forms. A high number suffered from ePVH without signs of disturbances in the pulmonary vascular bed (not out-of-proportion). A similarly high proportion of ePVH was found in a scleroderma collective by Saggard with 21% of patients (n = 54, resting PH was excluded) [15]. This underlines the need to collect more information on exercise pulmonary hemodynamics during RHC, as echocardiography would probably misclassify some patients since no PAWP can accurately be determined. None of the 44 scleroderma patients in this collective had postcapillary PH at rest, but a remarkable 1/3 had ePVH. This might explain why these patients were symptomatic with dyspnea and thus scheduled for RHC. However, the course of this entity and the best treatment options need to be better defined. Further observational and treatment studies will show if the classification by exercise hemodynamics is able to separate patients according to treatment response and outcome.

Our report on exercise RHC in real life illustrates the difficulties of daily clinical practice to classify patients. Too little is known about the exercise PH forms and their clinical course or benefit of treatment with PAH-specific therapy. Can an elevated mPAP> 30 mmHg at maximal exercise of 20 Watt in a symptomatic patient with scleroderma be compared to a similar absolute mPAP increase in a healthy patient at 150 Watt? In our and others opinion the answer would be no [7]. Should we treat ePAH or wait until PAH will develop years later? What does postcapillary out-of-proportion PH during exercise signify? Our real-life data show that the classification of patients with exertional dyspnea into PH classes or subcategories of formerly labelled borderline PH or exercise PH is difficult and more a continuum than clearly distinguished groups. We hope that with future prospective studies more insight can be gathered to this clinically important issue.

Performing maximal symptom-limited exercise RHC in sometimes highly symptomatic PH patients was absolutely safe in our collective, as we did not encounter any adverse events. As others, we believe that in experienced hands this diagnostic tool is safe and can be performed in clinical routine in the diagnostic work-up of patients with suspicion of PH [16, 17]. However, a problem in clinical practice and research is that no standardized protocols for exercise RHC testing are available. Important issues in performing exercise RHC are the type of exercise (dynamic vs. static), the increase in work load (linear, step-wise) and the body position during exercise. We used a completely supine position with a bicycle fixed at the end of the patient bed. In this position, subjects will perform considerably less work load compared to up-right cycling. However, the supine position has the advantage of a similar and calm thorax position for measurements. In general, it is accepted that exercise should rather be dynamic to avoid increases in systemic resistance and that intrathoracic pressure swings should be carefully reduced due to their effects on the pressure transducers in the pulmonary vasculature [18]. Therefore, isotonic arm exercise as it is performed in some catheter laboratories, should be discouraged [19].

Limitations of our study are, apart from the single-center experience and the retrospective design, the rather small number of patients in the different groups and the relatively short follow up with very few events, which did not allow estimates on prognosis. The patients in our collective where slightly overweight (mean BMI 25.1 kg/m^2^), which corresponds to the Swiss general population and other PH-collectives published. Intrinsically to the trial design, we do not have a healthy matched control group and we were thus limited to compare symptomatic patients according to their hemodynamic profile. Therefore, insight in normal physiological response is limited.

## Conclusion

In this real-life experience in symptomatic patients undergoing exercise RHC for suspected PH, we found that exercise RHC is safe without any adverse events. Patients with diseases known to be associated with PH, such as scleroderma, were found in all hemodynamically defined groups, and this underscores the importance of RHC in the diagnosis of PH. The facts that the vast majority of patients with mPAP-values between 20 and 24.9 mmHg at rest develop exercise PH and that patients with resting PH are older strongly argue for exercise PH as a precursor of resting PH. Whether an earlier treatment start in patients with exercise PH (especially ePAH) would stabilize the disease should be addressed in future studies.

## Abbreviations

DPG: Diastolic pulmonary gradient (diastolic PAP – PAWP)
ePAH: Exercise precapillary pulmonary hypertension
eoPVH: Exercise postcapillary out-of-proportion pulmonary hypertension
ePVH: Exercise postcapillary pulmonary hypertension
mPAP: Mean pulmonary artery pressure
NYHA: New York Heart Association
PAH: Pulmonary arterial hypertension
PVH: Postcapillary pulmonary hypertension
PAWP: Pulmonary artery wedge pressure
PH: Pulmonary hypertension
RHC: Right heart catheterization
TPG: Transpulmonary gradient (mPAP – PAWP)
WHO: World Health Organization.
